# GDF‐15 blockade: A multi‐directional approach to potentiate cancer immunotherapy and alleviate cancer cachexia

**DOI:** 10.1002/ctm2.70280

**Published:** 2025-03-30

**Authors:** Ignacio Melero, Kathrin Klar, Eugen Leo

**Affiliations:** ^1^ Clinica Universidad de Navarra CIMA, IDISNA and CIBERONC Pamplona, Spain, and Nuffield Department of Medicine, University of Oxford Oxford UK; ^2^ Clinical Development CatalYm GmbH Martinsried Germany

**Keywords:** cancer immunotherapy, cachexia, GDF‐15, visugromab

## 1 INTRODUCTION

**FIGURE 1:**
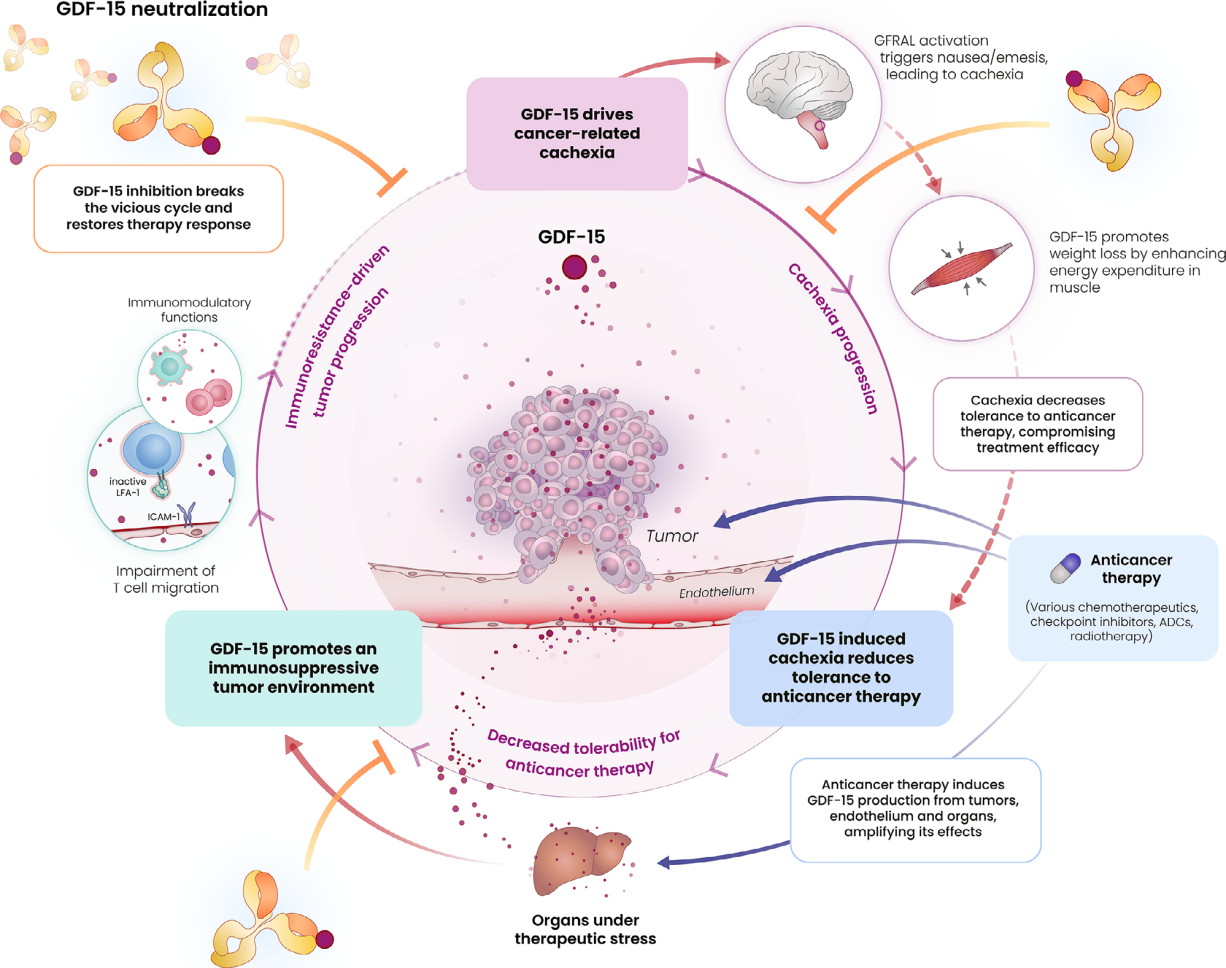
The ‘vicious cycle’ of GDF‐15 activity in subjects with cancer. This schematic illustrates the multi‐directional role of growth differentiation factor‐15 (GDF‐15) in promoting resistance to cancer (immuno‐)therapy and cancer‐related cachexia. GDF‐15 creates an immunosuppressive tumour microenvironment by suppressing T cell migration and ‐function, contributing to resistance to cancer (immuno‐)therapy and tumour progression. Tumour‐ (and therapy‐) induced GDF‐15 triggers cachexia by activating GFRAL signalling in the brain, leading to nausea, emesis and anorexia that causes weight loss and muscle wasting. This leads to reduced tolerance to anticancer therapy and further compromises effective cancer treatment. Anti‐cancer therapies in turn (e.g. platinum‐based drugs, antibody‐drug conjugates, checkpoint inhibitors, and radiotherapy) induce GDF‐15 overexpression from tumours, the endothelium, and various organs, further amplifying its deleterious effects. GDF‐15 neutralization breaks this vicious cycle at several key points and can help restore response to and tolerability of anticancer (immuno‐)therapy, thereby improving treatment outcomes.

Metastatic solid tumours remain a major challenge in clinical medicine, demanding innovation with novel treatment approaches and new synergistic combinations. Immunotherapy with immune checkpoint inhibitors (ICIs) targeting PD‐1 and PD‐L1 has improved the treatment outcome for numerous tumour types in a groundbreaking way. However, resistance to immunotherapy is very frequent and ultimately impacts the vast majority of patients treated, often resulting in fatal outcome. Similarly, in advanced cancer patients cachexia is a frequent event. This is a serious debilitating syndrome characterized by severe muscle wasting, weight loss and systemic metabolic dysfunction which associates with a dismal prognosis. Cachexia further worsens clinical outcome in cancer patients and can prevent ability to continue on therapy. In this context, our recent study published in Nature, titled “Neutralizing GDF‐15 can overcome anti‐PD‐1 and anti‐PD‐L1 resistance in solid tumours,” demonstrates the multifaceted roles growth differentiation factor 15 (GDF‐15) plays and provides initial data on a promising novel therapeutic strategy to counteract immunotherapy resistance and cachexia.^1^ Here, we discuss the potential benefits of GDF‐15 blockade as an emerging approach to both potentiate cancer immunotherapy and simultaneously mitigate cancer cachexia, highlighting recent advancements and their potential future clinical implications (Figure 1).

### 1.1 GDF‐15's role in tumour immune evasion

GDF‐15, a stress‐induced cytokine belonging to the transforming growth factor‐beta (TGF‐β) superfamily, is the most overexpressed cytokine in solid tumours^2^ and has recently been identified as a pivotal immunosuppressive factor within the tumour microenvironment (TME). In our study, integrative high‐throughput pan‐cancer immune‐transcriptomics analyses revealed significant correlations between GDF15 mRNA and immune signatures reflecting an immunosupressed TME in non‐small cell lung cancer (NSCLC), urothelial cancer (UC), and hepatocellular cancer (HCC), among others. Recent studies have demonstrated that tumour‐derived GDF‐15 can impair lymphocyte function‐associated antigen‐1 (LFA‐1)‐dependent T cell recruitment, thereby hindering effective immune surveillance and facilitating tumour immune escape.^3^ Similarly, placental GDF‐15 overexpression during the early phase of pregnancy appears to suppress an unwarranted immune reaction against the semi‐allogeneic foetus. Preclinical mouse models indicated that neutralization of GDF‐15 improves T‐cell infiltration and enhances responses to immune checkpoint inhibitors, particularly anti‐PD‐1/PD‐L1 therapy. Mechanistically, GDF‐15 blockade increases the trafficking of cytotoxic CD8+ T cells into tumours while simultaneously reversing an immunosuppressive milieu characterized by regulatory T cells (Tregs) and dampened dendritic cell activity.^4^

These effects underscore the potential of GDF‐15 blockade to sensitize resistant tumours and further potentiate response to checkpoint inhibitors, providing new avenues for combination immunotherapy.

### 1.2 Enhancing cancer immunotherapy response in solid tumour patients

In light of these findings, we investigated in our first‐in‐human trial if GDF‐15 blockade by visugromab could revert primary and secondary resistance to anti‐PD1/PD‐L1 treatment in ICI relapsed/refractory solid tumour patients.^1^ Only patients who had exhausted all available treatment options with tumours that had directly progressed on or after initial response had relapsed on continuously ongoing ICI were enrolled.

As demonstrated in sequential tumour biopsies visugromab treatment substantially increased T cell infiltration, T cell proliferation, granzyme B expression in cytotoxic T cells and interferon‐γ‐related signalling in the tumour. This was maintained or even further enhanced when adding nivolumab after the upfront, single‐cycle monotherapy phase.

In this highly refractory patient population, GDF‐15 blockade with visugromab in combination with an ICI achieved objective remissions in 14%–19% of patients with NSCLC, UC and HCC. Specifically noteworthy was the duration and depth of the responses achieved. More than half of the patients responding obtained a deeper response as per RECIST 1.1 categories (SD, PR, CR) than on their initial approved checkpoint inhibitor treatment regimen, which quite often had included chemotherapy. In addition, more than half of the responders achieved either a confirmed complete or a confirmed complete metabolic response (as per PET‐CT). This suggests that GDF‐15 blockade can significantly potentiate anti‐tumour immune response and may broaden, deepen and prolong checkpoint inhibitor activity significantly, up to complete response level.

### 1.3 GDF‐15 and cancer cachexia: Another important mechanistic link

Beyond its immunosuppressive role, GDF‐15 is a key driver of cachexia, a complex metabolic syndrome associated with loss of muscle mass, anorexia, chronic inflammation and insulin resistance.^5^, ^6^, ^7^ Cachexia affects up to 80% of advanced cancer patients and is associated with increased mortality and poor quality of life due to metabolic disturbances and increased systemic inflammatory response and weight and muscle mass loss.^8^, ^9^ Patients with advanced cancer exhibit significantly higher levels of GDF‐15 in their serum which correlates with weight loss, body mass index reduction and lipid metabolism dysregulation (reviewed in Reference [10]).

### 1.4 Blocking GDF‐15 to overcome cancer cachexia

Unlike conventional nutritional support, which often fails to counteract the catabolic pathogenesis of cachexia, GDF‐15 inhibition has the potential to directly target one of its underlying pathophysiologic causes. Preclinical models suggest that blocking GDF‐15 not only prevents further weight loss but may also recover physical activity in cachectic subjects.^11^, ^12^ Various pharmacologic approaches have been undertaken to treat or prevent cachexia. In September 2024, randomized phase 2 data for the anti‐GDF‐15 antibody ponsegromab were published in the New England Journal of Medicine demonstrating its potent activity to alleviate cancer‐related cachexia.^13^ Ponsegromab treatment at doses of up to 400 mg Q4W was very safe and led at the highest dose to significant weight gain (averaging > 5% in first 12 weeks) and improved physical activity and quality of life in patients undergoing standard‐of‐care anti‐cancer therapy substantially. Long‐term follow‐up (up to 1 year treatment) is still pending but expected to be reported in the second half of 2025. Various other GDF‐15 targeting compounds have now entered the field of cachexia‐treatment following ponsegromab, reflecting the strong interest in the field. It still remains to be elucidated though, if treatment of cachexia with GDF‐15 blockade will significantly improve outcome of cancer therapy and overall survival. Initiation of a phase 3 trial with ponsegromab has been announced for the second half of 2025.

Of important note, many highly effective chemotherapeutics (e.g. cis‐ and carboplatin) and targeted cytotoxics (e.g. antibody drug conjugates) currently indicated in first‐ and second‐line treatment of advanced solid tumours are known to induce tissue‐ and/or tumoral expression and secretion of GDF‐15.^14^, ^15^ This effect may contribute on top of the already existing tumoral GDF‐15 overexpression and secretion to the induction of nausea, anorexia and cachexia. It should also further enhance immunosuppression and resistance to cancer immunotherapy. This illustrates the vicious cycle GDF‐15 is creating: reducing tolerability to therapy, inducing cancer immunotherapy resistance and promoting physical decay via cachexia.

### 1.5 Future perspectives for GDF‐15 blockade in cancer

GDF‐15 blockade can potently prevent/reverse a key resistance mechanism for cancer immunotherapy and treat cachexia. In addition, it may improve the tolerability of standard‐of‐care regimens by reducing nausea and emesis. This naturally represents a tempting novel mult‐directional therapeutic concept and deserves further clinical investigation.

First‐ and second‐line therapies may benefit substantially by the addition of GDF‐15 blockade. Apart from being impaired already by GDF‐15 expression of the tumour (specifically anti‐PD1/‐L1), many approved therapeutics in these settings (including key chemotherapeutics, ADCs and ICIs) further fuel GDF‐15 overexpression/release and thereby can enhance immunotherapy resistance and cachexia. Hence, GDF‐15 blockade could become an important pillar of future anticancer combination therapy in early lines of treatment where these agents are mostly employed.

Ongoing and planned clinical trials will be evaluating the safety and efficacy of GDF‐15 neutralizing antibodies further with regard to cancer immunotherapy and cachexia and will read out in the coming years. Randomized visugromab trials in patients suffering from UC, non‐squamous NSCLC and HCC in early lines of treatment are already ongoing or will start later in 2025.

Despite these initial promising data, several challenges remain. Optimal dosing and combination strategies with immunotherapy as well as other standard‐of‐care modalities will require dedicated further exploration. Furthermore, the identification and validation of predictive biomarkers of GDF‐15‐driven immunosuppression and cachexia could help to stratify for patients who would benefit most from this therapeutic approach.

In conclusion, recent publications such as ours underscore the important role of GDF‐15 in cancer immunosuppression, resistance to immunotherapy and cachexia induction. Targeting GDF‐15 represents a promising strategy to both enhance the efficacy of cancer immunotherapies and alleviate cancer‐associated cachexia. As research progresses, integrating GDF‐15 inhibitors into clinical practice could offer substantial improvements. In our opinion, GDF‐15 blockade could play a critical role to overcome resistance to CPI immunotherapy as well as in the overall improvement of the quality of life of cancer patients. Further investigations into the molecular mechanisms and potential combinations with other therapies will be crucial to translate these findings into tangible clinical benefits.

## AUTHOR CONTRIBUTIONS

I. Melero, K. Klar and E. Leo prepared jointly the manuscript.

## CONFLICT OF INTEREST STATEMENT

I. Melero is principal investigator of the trial 1 and has served as a consultant to Catalym GmbH. K. Klar and E. Leo are employees of Catalym GmbH, Martinsried, Germany, a biotechnology company developing the anti‐GDF15 antibody visugromab.

## ETHICS STATEMENT

The authors declare human ethics approval does not apply for this manuscript.
